# Highly Sensitive Whole-Cell Mercury Biosensors for Environmental Monitoring

**DOI:** 10.3390/bios14050246

**Published:** 2024-05-13

**Authors:** Dahlin Zevallos-Aliaga, Stijn De Graeve, Pamela Obando-Chávez, Nicolás A. Vaccari, Yue Gao, Tom Peeters, Daniel G. Guerra

**Affiliations:** 1 Laboratorio de Moléculas Individuales, Laboratorios de Investigación y Desarrollo, Facultad de Ciencias e Ingeniería, Universidad Peruana Cayetano Heredia, Lima 15102, Peru; dahlin.zevallos.a@gmail.com (D.Z.-A.); pamela.obando@upch.pe (P.O.-C.); nicolas.arias.vaccari@gmail.com (N.A.V.); 2 Open BioLab Brussels, Erasmushogeschool Brussel, Laarbeeklaan 121, B-1090 Jette, Belgium; 3 Archaeology, Environmental Changes and Geo-Chemistry (AMGC), Vrije Universiteit Brussel, Pleinlaan 2, B-1050 Brussels, Belgium; yue.gao@vub.be

**Keywords:** whole-cell biosensor, mercury detection, artisanal gold mining, environmental monitoring, bioavailable heavy metals, MerR, Tn501

## Abstract

Whole-cell biosensors could serve as eco-friendly and cost-effective alternatives for detecting potentially toxic bioavailable heavy metals in aquatic environments. However, they often fail to meet practical requirements due to an insufficient limit of detection (LOD) and high background noise. In this study, we designed a synthetic genetic circuit specifically tailored for detecting ionic mercury, which we applied to environmental samples collected from artisanal gold mining sites in Peru. We developed two distinct versions of the biosensor, each utilizing a different reporter protein: a fluorescent biosensor (Mer-RFP) and a colorimetric biosensor (Mer-Blue). Mer-RFP enabled real-time monitoring of the culture’s response to mercury samples using a plate reader, whereas Mer-Blue was analysed for colour accumulation at the endpoint using a specially designed, low-cost camera setup for harvested cell pellets. Both biosensors exhibited negligible baseline expression of their respective reporter proteins and responded specifically to HgBr_2_ in pure water. Mer-RFP demonstrated a linear detection range from 1 nM to 1 μM, whereas Mer-Blue showed a linear range from 2 nM to 125 nM. Our biosensors successfully detected a high concentration of ionic mercury in the reaction bucket where artisanal miners produce a mercury–gold amalgam. However, they did not detect ionic mercury in the water from active mining ponds, indicating a concentration lower than 3.2 nM Hg^2+^—a result consistent with chemical analysis quantitation. Furthermore, we discuss the potential of Mer-Blue as a practical and affordable monitoring tool, highlighting its stability, reliance on simple visual colorimetry, and the possibility of sensitivity expansion to organic mercury.

## 1. Introduction

Mercury is ubiquitous in nature but its distribution has significantly increased in recent years due to anthropogenic activities such as coal combustion, mining, and agriculture [1,2]. This metal is well known for its toxicity even at low concentrations. Mercury exposure can cause fatal effects on the kidney, nervous system, and brain, and can lead to the development of Minamata disease, a neurological syndrome caused by severe mercury poisoning [3,4,5]. Given its toxicity, mercury exposure has become a potentially global health problem. To effectively control pollution, it is crucial to monitor mercury levels in the environment [6]. However, conventional quantitative techniques like atomic fluorescence spectrometry and atomic absorption spectrometry require expensive equipment and specialized training for their use [7]. Moreover, the high-risk areas are geographically extensive, which greatly hinders the efforts of environmental control authorities. Furthermore, as these areas are typically rural and plagued by illegal practices, they are distant from specialized personnel and high-tech development in general [8]. Therefore, there is a need to develop a cost-effective technique for mercury monitoring to protect human health and the environment through decentralized accessible strategies.

Whole-cell biosensors (WCBs) hold immense promise as future tools for environmental pollutant detection. Microorganisms, remarkably adaptable, thrive in diverse environments, even harsh ones. They possess genes that grant resistance to various toxins. As a consequence of the significant metabolic cost linked to these resistance mechanisms, evolution has equipped them with highly sensitive and specific regulatory circuits. Thanks to advancements in synthetic biology, the components of these natural systems can now be incorporated into synthetic gene circuits.

Mercury-sensitive WCBs are typically based on a transcription factor called MerR as the core sensing element. MerR recognizes a specific DNA sequence known as the operator. This operator is an integral part of a promoter [9,10], where the RNA polymerase binds to initiate the transcription of genes that confer resistance to metal toxicity. Importantly, this promoter is not competent for transcription, and it is only in the presence of mercury ions that MerR, bound to the promoter–operator sequence, responds with a conformational change that optimizes the DNA position for recruiting active RNA polymerases [11]. In nature, MerR regulates its own expression [9,12,13]. In engineered WCBs, MerR regulates reporter proteins which can be chromoproteins or enzymes such as luciferases or beta-Galactosidase [14,15].

Some of these engineered systems have achieved remarkable sensitivity and low limits of detection in research settings. Furthermore, the literature increasingly demonstrates the versatility of potential WCB designs. While some studies focus on improving input characteristics like limit of detection, sensitivity, dynamic range, and specificity [16,17], others prioritize user-friendly outputs with strong signals [18,19,20]. These advancements are paving the way for the development of WCBs as practical tools for environmental monitoring.

However, many innovations face challenges in real-world application. These limitations include restrictive GMO regulations [21], portability issues, and the inherent difficulty of maintaining living cells. Furthermore, while WCBs show promise in the lab, their adoption for sensitive applications like environmental monitoring and health necessitates extensive validation with real-world environmental samples. This gap between lab capabilities and real-world needs underscores the critical need for refinement, enhancement, and the involvement of the research community alongside policymakers and local authorities. In this complex context, tailoring sensors to specific environments might be necessary, making local development and testing crucial. Unfortunately, researchers in developing countries, where access to standard analytical techniques is most limited, have not been extensively involved in these advancements.

With the long-term goal of developing a user-friendly, locally produced biosensor, we designed a simple circuit using readily available gene parts. We wished to explore if the simplest approach could produce adequate sensitivity parameters, without necessarily resorting to the most recent strategies of advanced bioengineering such as transcriptional amplifiers or the engineering of metal transporters [22]. We developed two versions of our biosensor: The first uses a fluorescent reporter (RFP), which allowed a full characterization of its functioning and the interdependence of the signal and cell density during growth. The second version uses a colorimetric reporter (AmilCP), visible by the naked eye, that we coupled to a low-cost custom-made camera setup. We tested our WCB with real samples from currently operating artisanal gold mining sites in Madre de Dios, Peru. The results are promising and encourage further development.

Despite these encouraging results, we observed that the rate of bacterial growth affects the output signal and thus may interfere with the detection of ionic mercury in environmental samples. These observations, which result from the general constraints of resource allocation to native versus heterologous functions [23], underscore the importance of fully characterizing WCBs as living cultures. This entails following reliable and reproducible protocols of microbiological manipulation in addition to directly measuring practical parameters such as LOD, sensitivity, and dynamic range.

## 2. Materials and Methods

### 2.1. Bacterial Strains, Plasmids, Media, and Reagents

*E. coli* DH5α [*fhuA2* ∆*(argF-lacZ)U169 phoA glnV44 Φ80* ∆ *(lacZ)M15 gyrA96 recA1 realA1 endA1 this-1 hsdR17*] was used as the host cell for DNA manipulation. Other strains of *E. coli* (BL21 start DE3 plys, Rosseta, BL21 (DE3), BL21 Codon Plus (DE3) RIL, and SoluBL21) were evaluated as chassis for the optimal functioning of the biosensors (Figure S1). The plasmid pUC57, which carries ampicillin (AMP) resistance, was used as the vector for both versions of the mercury WCB. For DNA cloning processes, *E. coli* cells were grown in Lauri Bertani (LB) medium. The characterization assays were conducted in M9 minimal medium (Sigma-Aldrich, St. Louis, MO, USA), supplemented with 0.24% (*w*/*v*) glucose, 0.2% cas-amino acids, 2 mM MgSO_4_, and 1 mM CaCl_2_. Mercury (II) bromide (HgBr_2_) was used as the inducing agent. Both HgBr_2_ and AMP were purchased from Sigma-Aldrich in solid form, dissolved in ultrapure water, and filtered through 0.22 µm syringe filters. The HgBr_2_ solutions were diluted as specified in Supplementary Table S1.

### 2.2. Plasmid Circuit Construction

The sequences for the *merR* gene and its promoter–operator were obtained from the transposon Tn501 from *Pseudomonas aeruginosa* plasmid pVS1 (GenBank: Z00027.1). These parts were combined in silico with the P429 synthetic promoter [16], the *amilCP* gene (BioBrick BBa_K592009) [24], and two transcription terminators (lambda TR2 and BioBrick BBa_B0014) to design the biosensor circuit described in Section 3.1. The circuit was synthesized and cloned into the pUC57 vector by GeneScript Biotech, resulting in the creation of the pUC-Mer-Blue plasmid. Subsequently, the *amilCP* gene was replaced by *rfp* reporter gene [25] through traditional cloning, resulting in the formation of the pUC-Mer-RFP plasmid. All sequences used in this study are summarized in Supplementary Table S2.

### 2.3. Reporter Expression Assay

To activate the biosensor prior to the assays, a single colony of *E. coli* harbouring a sensor plasmid (pUC-Mer-Blue or pUC-Mer-RFP) was taken from an LB-agar Petri dish and grown overnight in M9 medium with 50 μg/mL ampicillin at 37 °C with 220 rpm constant shaking. At the start of each assay, the bacterial culture was diluted to the indicated density.

Measurements of 200 μL microcultures were taken with a microplate reader (Tecan Infinite 200 Pro, Männedorf, Switzerland) every 15 min for 16 h at 37 °C with fast shaking. All presented conditions were measured in three independent wells. The fluorescence of RFP was measured in arbitrary fluorescence units (AFU) using the following settings: excitation 570 nm, emission 615 nm. Bacterial growth was determined by optical density at 600 nm (OD600).

For each induction assay in a microplate reader, 5 μL of a HgBr_2_ solution of the adequate concentration was added to 195 μL of culture to reach the annotated final concentrations. To assess the effect of induction with mercury at different times, the experiment described in Section 3.3 started with a dilution of 1:1000 from an overnight culture, resulting in an initial OD600 of 0.002. Subsequent experiments were run with an initial OD600 of 0.050.

To quantify the accumulation of the reporter protein AmilCP, cultures were initiated similarly, but with 10 mL volumes instead of 200 μL. Induction was triggered by adding 10 µL of an HgBr2 solution to reach the concentration specified in the text. The cultures were then incubated at 37 °C for 16 h with vigorous shaking at 220 rpm. To harvest the cells, 1 mL aliquots of culture were loaded into 1.5 mL microtubes and centrifuged at high speed. This process was repeated five times to obtain a large pellet. Following the procedure described by Liljeruhm et al. in 2018 [26], photographs of the pellets in 1.5 mL microtubes were captured. These microtubes were positioned in a custom-built set-up ensuring consistent illumination and camera positioning. A representative nine-pixel area was selected from the image of each pellet to calculate the average colour intensity across the three red, green, and blue (RGB) channels. Colour intensity was computed as the Euclidean distance (*E*) in the RGB space relative to a white reference pellet (non-transformed *E. coli* cells), as previously described [26], using the following equation:

(1) $$E=\sqrt[{2}]{{\left(R_{sample}-R_{ref}\right)}^{2}+{\left(G_{sample}-G_{ref}\right)}^{2}+{\left(B_{sample}-B_{ref}\right)}^{2}}.$$

Here, *R_sample_*, *G_sample_*, and *B_sample_* represent the average colour intensities from each RGB channel in the nine-pixel area of the pellet image from the sample of the biosensor, while *R_ref_*, *G_ref_*, and *B_ref_* denote the corresponding values for a reference pellet of non-transformed *E. coli*.

### 2.4. Environmental Samples from Madre de Dios, Peru

Samples of water (knee-high) and samples of 50% water-sediment mix (pond bottom) were collected from two active artisanal gold mining sites: Laberinto district and Isla de los Monos, in Puerto Maldonado, Madre de Dios, Peru. In total, seven samples were collected: four from active mining ponds; two from old, inactive mining ponds; and one from the amalgam reaction bucket. The latter is representative of the residues that artisanal miners discard into rivers or mine ponds after the amalgamation process is complete. All samples were centrifuged at 4000 rpm for 10 min to isolate the aqueous fractions, which were then passed through 0.22 µm syringe filters to obtain sterile dissolved water samples.

Before ICP-MS analysis, hydrochloric acid (HCl) (Fisher, Brussels, Belgium, analytical grade, 37%) was added to the dissolved samples, achieving a final concentration of 5% HCl in samples. Afterwards, these samples were transported and analysed for Hg concentrations by using Sector Filed-ICP-MS (Thermo, Bremen, Germany, Element 2) at the research unit of Archaeology, Environmental and Geochemistry of Vrije Universiteit Brussel (VUB).

For analysis with both Mer-Blue and Mer-RFP, 5 mL aliquots of each sterile-filtered (0.22 µm) sample were mixed with 4.5 mL of 2X M9 medium supplemented with glucose and casamino acids. This resulted in a total volume of 9.5 mL per sample. We then inoculated each sample with 0.5 mL of an overnight WCB culture that had been pre-resuspended in fresh medium. This step diluted the samples by half and the culture by a factor of 20, leading to an initial OD600 of 0.05.

### 2.5. Data Analysis

#### 2.5.1. Relative Concentration of the Reporter Protein

The fluorescence of RFP was normalized by the density of the culture, according to the following expression:

(2) $$\frac{Fluo}{OD600}=\frac{{Fluorescence}_{sample}-{Fluorescence}_{blank}}{{OD600}_{sample}-{OD600}_{blank}},$$

where ${Fluorescence}_{blank}$ and ${OD600}_{blank}$ represent the fluorescence and absorbance of the culture medium, respectively.

#### 2.5.2. Promoter Activity

The promoter activity is defined here as the protein synthesis rate normalized by the density of the culture, denoted as f(t), and calculated through the following expression [27]:

(3) $$f(t)=\frac{1}{OD600}\frac{dfluo\;}{dt}=\frac{1}{{OD600}_{n}}\;\;\;\left(\frac{{Fluo}_{n+1}-{Fluo}_{n}}{t_{n+1}-t_{n\;}}\right).$$

#### 2.5.3. Dose–Response Curves

The maximum protein synthesis rate in the exponential phase, denoted as $F_{max}$, where $d(f\left(t\right))/dt=0$, was determined for each concentration of the inducer to evaluate the dose–response relationship [28]. The resulting data were plotted in a dose–response graph (described in Section 3.4 and Section 3.5) and fitted to a Hill function. This model aims to describe the relationship between the input and output signals of a genetic circuit at steady state, given by the following form:

(4) $$F_{max}=\psi_{min}+\psi_{max}\left(\frac{{\left[I\right]}^{h}}{{\left[I\right]}^{h}+K_{I}^{h}}\right),$$

where $F_{max}$ is the steady-state synthesis rate for the reporter protein under the control of the inducible promoter retrieved from Tn501; $\psi_{min}$ is the baseline protein synthesis rate from the same promotor without induction; $\psi_{max}$ is the maximum protein synthesis attainable by the system; I is the concentration of inducer (i.e., ionic mercury, ${Hg}^{2+}$); h is the Hill coefficient; and $K_{I}^{h}$ is the Hill constant which equals the concentration of external input [I] that yields a half-maximal response.

The experimental data were fitted using the Nonlinear Least Squares method, implemented in the R programming language.

#### 2.5.4. Calculation of Linear Range

To determine the range of concentrations over which the biosensor responds linearly to changes in the inducer concentration, we employed a logarithmic transformation of the variables $\left[{Hg}^{2+}\right]$ and $F_{max}$. This was followed by a linear regression analysis to establish the following equation:

(5) $$\ln\left(F_{max}\right)=m\cdot \ln\left[{Hg}^{2+}\right]+y_{0}.$$

Here, m is the slope of the linear function, and $y_{0}$ is the intercept on the y-axis when $\ln\left[{Hg}^{2+}\right]=0$. We then applied this equation to estimate the concentration of ionic mercury in environmental samples, similar to the approach described in [29].

#### 2.5.5. Calculation of Limit of Detection (LOD)

LOD is defined as the smallest concentration that can be detected and distinguished from the baseline signal. It was calculated using the following equation [30]:

(6) $$\ln\left(LOD\right)=3.3\;\sigma_{0}/m,$$

where $\sigma_{0}$ is standard deviation of the linear regression, and m is the slope of Equation (4).

#### 2.5.6. Bacterial Growth

To analyse possible differences in growth when the biosensor is exposed to different concentrations of ionic mercury, the culture’s density, calculated as ${OD600}_{sample}-{OD600}_{medium}$, was used to compute the culture’s growth rate using the following equation:

(7) $$dOD600/dt=\frac{{OD600}_{n+1}-{OD600}_{n}}{t_{n+1}-t_{n\;}}.$$

The maximum growth rate observed in each culture was then used to compare growth rates across different concentrations of mercury.

## 3. Results

### 3.1. Design of a Synthetic Genetic Circuit for Mercury Detection

**Figure 1:**
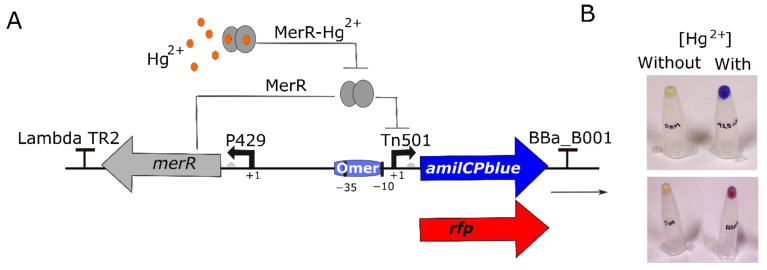
Design of synthetic genetic circuit to detect ionic mercury; (**A**) The schematic shows how this genetic circuit works. The expression of MerR sensor protein (MerR) is driven by constitutive promoter P429. MerR protein binds to operator mer (Omer), repressing the expression of the amilCP or rfp genes. In presence of Hg^2+^, MerR sensor binds to its cognate ligand (ionic mercury) and activates the expression of the reporter gene (amilCP or rfp). (**B**) Pellets of cells from overnight cultures in the presence or absence of 125 nM HgBr_2_.

In this study, we developed an *E. coli* WCB based on an uncoupled genetic circuit, meaning that the expressions of the transcription factor and the reporter protein originate from separate, divergent, and physically distant promoters. This circuit incorporates the MerR transcription factor from *Pseudomonas aeruginosa* as its core element, selected for its high specificity for ionic mercury [9,31]. To regulate the expression of the *merR* gene, we employed the P429 medium-strength synthetic promoter, which was previously used to achieve optimal concentrations of MerR protein [16]. As reporter proteins, we selected RFP and AmilCP proteins due to the absence of endogenous red fluorescence or blue colour in the host *E. coli* cells. Notably, the chosen excitation and emission wavelengths for RFP detection produced no autofluorescence from our cultures. Similarly, blue is a rare colour in biological systems, suggesting AmilCP will provide a striking contrast in diverse settings. We controlled the expression of the reporter genes using the native promoter from Tn501, which contains a mer operator (Omer) positioned between the −10 and −35 regions recognized by RNA polymerase [10]. To prevent unintended interference between the P429 promoter controlling the MerR constitutive expression and the promoter from Tn501 controlling the reporter’s inducible expression, we inserted a 500 bp sequence between them (Figure 1A). Additionally, restriction sites flanking each circuit element were included to facilitate future modifications (Supplementary Table S2).

### 3.2. Testing the Biosensor Circuit

The plasmids pUC-Mer-RFP and pUC-Mer-Blue were introduced into the *E. coli* DH5α competent cells. For the first analysis, both WCBs were cultured in the presence and absence of 125 nM ionic mercury. The initial results revealed significant red or blue staining in the pellet of cells obtained from the mercury-containing biosensor cultures, contrasting with the mercury-free samples (see Figure 1B).

On the other hand, the performance of a genetic circuit can vary significantly between different bacterial chassis, even if the same genetic circuit is used in each strain [32]. The results showed that *E. coli* DH5α served as the best host cell for our biosensor circuit (Figure S1). Henceforth, we will refer to the WCBs constituted by DH5a cells carrying the pUC-Mer-RFP and pUC-Mer-Blue plasmids as Mer-RFP and Mer-Blue, respectively.

### 3.3. Reporter Expression Induced at Various Phases of Bacterial Growth

**Figure 2:**
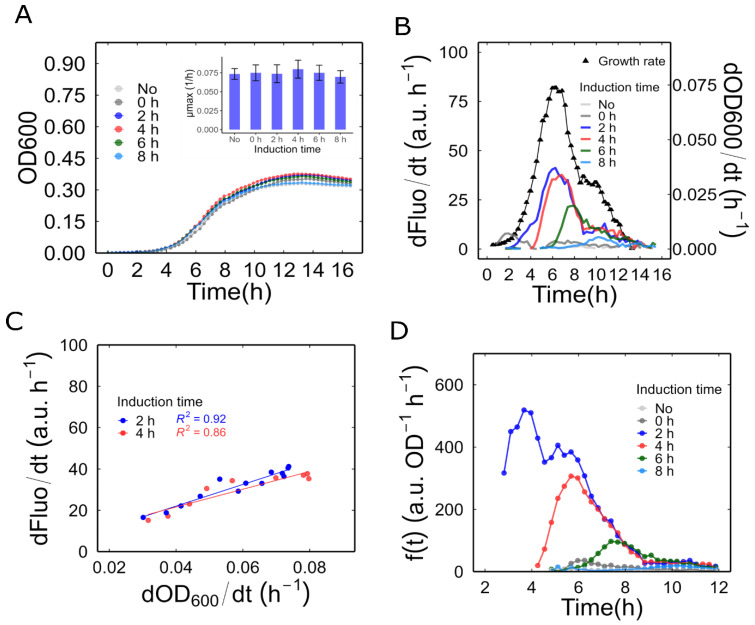
Dynamics of the inducible promoter behaviour during bacterial growth: (**A**) Bacterial growth of MerR-RFP biosensor induced at different times. The inner graph shows the maximum observable growth rate between different induction times. Error bars are ± one standard error. (**B**) Fluorescence synthesis rate of sample in different bacterial growth phases. The bacterial growth phase was determined by analysing the ln (OD600). The growth rates plotted in black correspond to the cultures induced at 2 h. The growth phases can be classified as follows: lag phase, from 0 to 2 h; exponential phase, from 2.5 to 8.5 h; and stationary phase, from 9 to 16 h. (**C**) Linear relationship between fluorescent protein synthesis rate and growth rate during the exponential phase. (**D**) Promoter activity in the exponential phase. All values are the means of three independent samples.

The subsequent analysis of WCB behaviour involved various phases of bacterial growth, starting at a low culture density. As outlined in the Materials and Methods section, the Mer-RFP biosensor was cultured starting at an OD600 of 0.002 and exposed to 50 nM Hg^2+^ (final concentration) at different induction times: 0, 2, 4, 6, and 8 h. Our analysis revealed that exposure to 50 nM Hg^2+^ at any given time did not produce significant differences in bacterial growth or maximum growth rate (Figure 2A).

Subsequently, we estimated the RFP synthesis rate by determining the change in fluorescence during various growth phases. Given that the OD600 in this experiment starts at very low values, normalizing the RFP synthesis rate and growth rate over OD600 resulted in noisy results that were difficult to interpret. Therefore, in this experiment, we estimated the promoter activity without normalization. In this manner, the change in fluorescence per unit time (dfluo/dt) and the growth rate (dOD600/dt) can be interpreted as the production rate and the growth rate of the culture as a whole, respectively. As depicted in Figure 2B, when mercury was introduced during the lag phase (induction at 0 h), the promoter was activated slowly. However, this activation was transient and was suppressed during the early exponential phase. When induced during the exponential phase (at 2 or 4 h), the reporter’s promoter was rapidly activated, revealing a linear relationship between the fluorescence production rate and the culture’s growth rate (Figure 2C). If the induction was performed later, closer to the stationary phase (at 6 and 8 h), promoter activity was also triggered, albeit to a lesser extent, as its decrease coincided with the decrease in growth rate. This response was shorter and consequently accumulated less signal compared to the response observed at 2 and 4 h of induction.

Finally, we calculated the promoter activity as indicated in the Materials and Methods section. This was carried out using only the exponential phase data, where the OD600 values are less noisy and allow for more realistic normalization than in the lag phase. As shown in Figure 2D, the rate of synthesis per unit of culture density (a proxy for production per cell) reached a significantly higher value after induction at 2 h compared to other induction times. The OD600 at this optimum induction time was 0.025 in the microplate reader (which corresponded to an OD600 of 0.050 in a 1 cm path length cuvette). Therefore, we selected this bacterial density as the starting point for further characterization of our biosensors.

### 3.4. Characterization of Mer-RFP

We profiled the dose response of Mer-RFP using microcultures with an initial OD600 of 0.05. These cultures were exposed to a range of final Hg^2+^ concentrations from 1 to 2000 nM. As expected, normalized fluorescence increased over time at all mercury concentrations, including the lowest (1 nM Hg^2+^). Interestingly, the response was not entirely smooth. We observed an initial burst of reporter production within the first hour, followed by a decrease and then a steady rise starting at around 2 h. This continued even as the cultures entered the stationary phase. We did not anticipate this rapid response within the first hour, given the RFP’s maturation time of close to 60 min. To investigate the mechanisms behind this initial burst, a faster maturing fluorescent protein, such as GFPmut3 (maturation time: 4 min), would be necessary.

**Figure 3:**
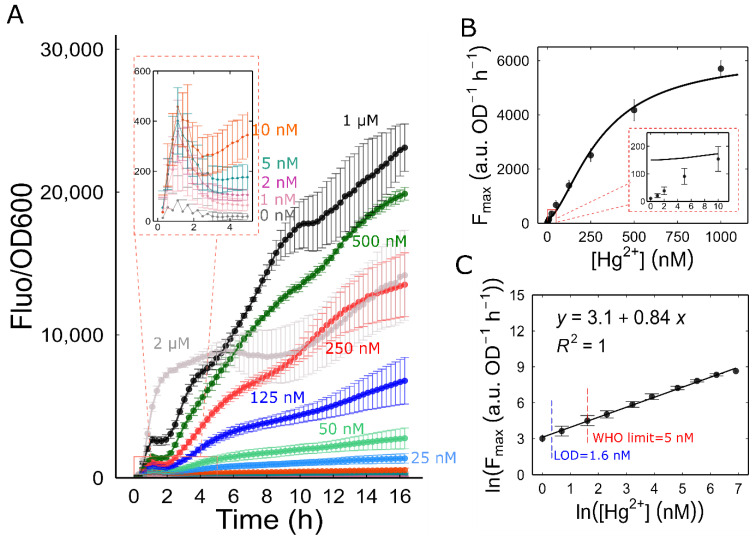
Characterization of Mer-RFP biosensor: (**A**) Fluorescence of Mer-RFP biosensor normalized by the density of the culture in response to different concentrations of ionic mercury. (**B**) The maximum promoter activity, $F_{max}$, for each concentration was used to generate a dose–response curve. The $F_{max}$ of the cultures with 2 μM HgBr_2_ concentration was excluded from the plot. The line represents a fitting to Hill function: $K_{I}^{h}=305\;nM$, $\psi_{min}=150$, $\psi_{max}=6000$, h=1.6. (**C**) The linear range of Mer-RFP biosensor. The dose–response curve was linearized by plotting the logarithms of the $F_{max}$ and Hg^2+^ concentration values. All symbols represent the means of 3 independent samples and the error bars indicate ± one standard error.

We observed that, at 2 μM Hg^2+^, the normalized fluorescence was lower than that obtained at 500 nM and 1 μΜ (Figure 3A). Additionally, at this concentr ation, the growth rate was negatively affected, resulting in a lower maximum growth rate than at other concentrations (Figure S2). This observation suggests that 2 μM Hg^2+^ is above a toxic threshold concentration, causing a decrease in both fluorescence and growth rate.

To accurately determine the biosensor’s linear range and limit of detection (LOD), we first calculated the promoter activity for each mercury concentration. We then used the maximum value of the protein synthesis rate ($F_{max}$) for each concentration as a representative point to create a dose–response curve (Figure 3B). Finally, the dose–response data were plotted using a natural logarithmic transformation of the Hg²⁺ concentration and the $F_{max}$. Our results showed that the biosensor response was linear within the range of 1 nM to 1 μM of Hg^2+^ ($\ln F_{max}=0.84\cdot \ln\left[{Hg}^{2+}\right]+3.1$). According to this linear regression, we determined the LOD to be 1.6 nM, which is well below the World Health Organization’s recommended limit of 0.001 mg/kg (5 nM, assuming water density of 1) for natural mineral waters [33] (Figure 3C).

### 3.5. Characterization of Mer-Blue

**Figure 4:**
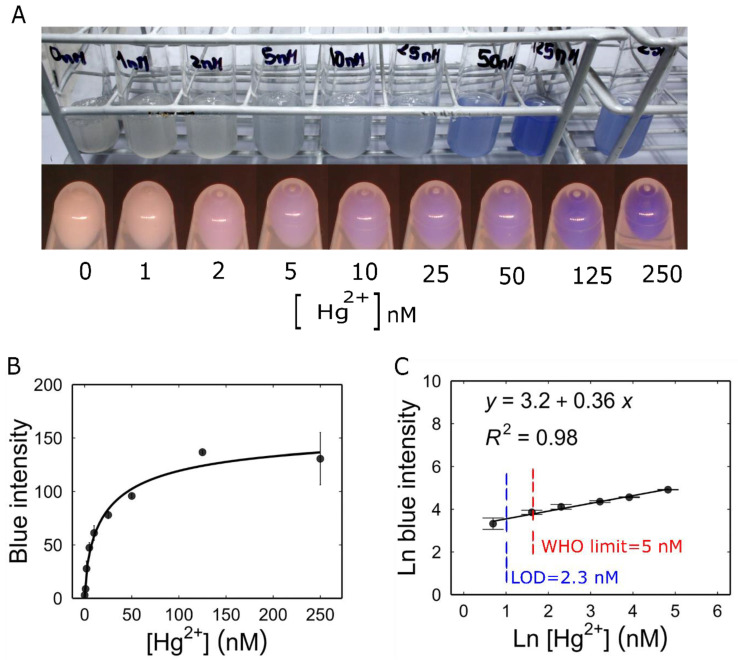
Characterization of Mer-blue biosensor: (**A**) Response of Mer-blue biosensor to increasing mercury concentrations, 16 h post induction. Top, biosensor cultures in M9 medium. Bottom, 5 mL of each culture was centrifuged, and photographs were taken using the custom-built camera setup. (**B**) Dose–response curve. Pellet pictures were analysed for colour intensity. The curve represents fitting to a Hill function: $K_{I}^{h}=23.67\;nM$, $\psi_{min}=0.9088$, $\psi_{max}=161.74$, h=0.6986. (**C**) Linearization of the dose–response curve. All values are the means of 3 independent samples and the error bars indicate ± one standard error.

Unlike the real-time measurement of RFP production, to characterize the Mer-Blue biosensor, the accumulation of AmilCP reporter protein was measured by quantifying the colour intensity at a single time point. As shown in Figure 4A, the biosensor allowed visual detection within the concentration range of 5 nM to 250 nM Hg^2+^ directly in the culture medium. Cells were harvested by centrifugation and the colour intensity in the pellets was measured from photographs, extending the range of detection down to 2 nM.

To calculate the biosensor parameters, the intensity of the blue colour measured for each concentration was used to create the dose–response curve (Figure 4B). We linearized the dose–response curve using the logarithmic transformation of both Hg^2+^ concentration and the colour intensity signal *E* defined in the Materials and Methods section. Our result showed that Mer-Blue had a linear range from 2 to 125 nM Hg^2+^. This relationship determined the following linear regression equation: $\ln E=0.36\cdot \ln\left[{Hg}^{2+}\right]+3.2$ (Figure 4C). Utilizing this linear regression, we determined the LOD to be 2.3 nM, which is also below the WHO-recommended limit for drinking water (0.001 mg/kg, or 5 nM) [33].

When the concentration of Hg^2+^ exceeded 250 nM, the accumulation of colour showed a drop. Although it was observed that this concentration of Hg^2+^ did not have a toxic effect on bacterial growth by itself, the pellet of the Mer-Blue biosensor was smaller than for other concentrations (Figure 3A). This suggests a detrimental interaction between the production of AmilCP and the exposure to mercury, possibly due to an antagonism between the synthesis of this reporter protein and stress-related proteins.

Consistent with observations from previous whole-cell biosensors utilizing the MerR transcription factor [34], Mer-Blue displayed minimal response to other metals. In tests with Ag, Cd, Cr, Fe, Ni, Pb, and Zn, none significantly increased the signal. Additionally, when a metal sample was co-administered with 100 nM Hg^2+^, it did not interfere with the signal output of Mer-Blue (Figure S3, Supplementary Materials).

### 3.6. Analysis of Environment Sample from Madre de Dios, Peru

To assess their functionality for environmental monitoring, we tested our Mer-RFP and Mer-Blue biosensors against samples extracted from artisanal gold mining sites. We collected seven samples from the surrounding areas of Puerto Maldonado, in the Peruvian region of Madre de Dios. As described in the Materials and Methods section, the samples were diluted to 50% with a mixture of 2x culture medium and a biosensor inoculum. Subsequently, after incubation and applying the linear equations from Figure 3C and Figure 4C, we utilized the biosensors’ responses to calculate the concentration of ionic mercury in each sample.

Our analysis (Table 1) showed undetectable levels of ionic mercury in most samples. This aligns with the chemical analysis, which revealed total mercury concentrations below the limits of detection (LODs) for both Mer-Blue and Mer-RFP. The highest mercury content was found in water–sediment samples from active mining ponds (2.72 and 2.37 nM Hg, respectively). However, after a two-fold dilution during sample preparation, these concentrations approached the lower LOD for Mer-RFP. Disappointingly, Mer-RFP did not detect any mercury in these samples. Even though the diluted concentrations (1.36 and 1.16 nM Hg) were close to the LOD (1.6 nM), they apparently fell just below the detection threshold.

In contrast, a sample from the amalgam reaction bucket contained significantly higher levels of ionic mercury. The Mer-RFP biosensor measured 52.3 nM Hg^2+^, while the Mer-Blue biosensor detected 45.9 nM Hg^2+^. Chemical analysis revealed a lower concentration of 25.13 nM Hg^2+^, but all values remained within the same order of magnitude (Table 1).

## 4. Discussion

In this study, we developed two versions of a whole-cell mercury biosensor, Mer-RFP and Mer-Blue, based on the MerR transcription factor and the native promoter–operator retrieved from a *P. aeruginosa* Tn501 transposon. Mer-RFP and Mer-Blue exhibit undetectable baseline expression of the reporter proteins and LODs below the WHO limit for drinking water, detecting from 1.6 nM Hg^2+^ to 2.3 nM Hg^2+^, respectively. Additionally, Mer-RFP exhibits a remarkably wide dynamic range with linearity from 1 to 1000 nM Hg^2+^, whereas the linear range of Mer-Blue covers from 2 to 125 nM Hg^2+^, thus demonstrating their potential applicability for a wide range of mercury concentrations.

Our biosensors exhibited some of the best performances when compared to previously reported mercury biosensors [16,22,29,31,34,35,36,37,38,39,40,41,42] (see Table S3, Supplementary Materials). This makes them promising candidates for developing effective tools for monitoring environmental samples. These results are encouraging, especially considering the circuit’s relative simplicity. This simplicity suggests that it could be readily enhanced through the innovative improvements recently introduced in the field of WCBs. A study achieved a remarkable improvement in the LODs for arsenic by lowering the expression of the sensing transcription factor repressor ArsR. This allowed lower amounts of the analyte to trigger the de-repression of the reporter promoter [22]. In that study, the lowered expression of MerR also improved the LOD for this metal; however, unlike ArsR, MerR needs to remain bound to DNA for reporter expression. In our study, we used a high expression of MerR through a medium-strength promoter (P429) in a high-copy plasmid. It is worth investigating whether lowering MerR expression could improve the LOD of our biosensors, considering theirs achieved a tenfold lower detection limit compared to ours. Unfortunately, fine-tuning transcription factor expression in a stable way might be challenging due to environmental noise and unpredictable sample contents. This is especially true considering the strong link between cell growth and protein production.

Aside from improvements at the level of the core regulation circuit, other enhancements may be introduced in the presentation of the cellular chassis and in the protocols for sample processing. For example, our sample protocol requires diluting them by half, which effectively doubles the limit of detection (LOD). We proceeded in this way aiming to perform the analysis of environmental samples in conditions that were as similar as possible to the conditions used for the analysis of the sensors. However, this dilution could be avoided if the growth conditions were less restrictive. The output signal is highly sensitive to the growth curve, as shown with Mer-RFP, where the induction time determines the reporter protein accumulation curve. Similarly, Mer-Blue shows a lower signal when growth is affected by high mercury concentrations. This interdependence between gene expression and the growth curve is inherent to bacterial physiology [23]. We believe this dependence might be the main reason why WCBs are not yet reliable enough for widespread environmental monitoring. Therefore, future developments should focus on decoupling the growth curve from sensitivity. An attractive strategy could be to reduce the burden of signal production by replacing the chromogenic output with an enzymatic one. Since a few enzyme molecules can theoretically produce unlimited product, reporter expression could be lowered. This would decrease the metabolic burden, potentially improving cell robustness and signal output. Another approach involves novel techniques for immobilizing freeze-dried cell-free extracts [43,44,45]. This strategy separates growth from sample presentation. Cells are grown under controlled laboratory conditions, then freeze-dried extracts with very high biomass are used for analysis. These extracts no longer require growth, simplifying the process.

In its current stage of development, the Mer-Blue biosensor may offer a cost-effective solution for performing quick screening for high mercury pollution in settings where microbiological manipulation is accessible while standard chemical analysis is not. Unlike its fluorescence-based counterparts, MerR-Blue utilizes a colorimetric reporter gene, enabling straightforward analysis via relatively simple microbiological manipulations. This eliminates the need for expensive plate readers with fluorimeter functions, replacing them with a low-cost DIY camera setup, a simple centrifuge, and a personal computer [16,20,29]. Importantly, Mer-Blue demonstrated remarkable stability across generations, retaining its sensitivity even after ten passages (Figure S4).

When attempting to analyse samples from diverse regions of Peru, such as Iquitos and Cajamarca, using Mer-Blue, we encountered instances where this biosensor did not grow, making it impossible to determine whether ionic mercury was present or absent (Figure S5). This evident drawback highlights the importance of exploring methods to minimize the impact of bacterial growth on the expression of reporter genes in WCB in order to produce reliable results in spite of varying conditions.

We evaluated the effectiveness of the biosensors using samples from artisanal gold mining in Madre de Dios, Peru. As elucidated in the Results section, only the sample from the reaction amalgam bucket exhibited mercury levels surpassing the WHO threshold by one order of magnitude. This finding indicates that a significant portion of metallic mercury (Hg^0^) added by the miner is transformed into its ionic form (Hg^2+^), enabling our biosensor to detect it. When the waste from the bucket is discarded into rivers or mining ponds, the concentration of ionic mercury decreases due to its dilution within a large mass of water. Thus, we observed low mercury concentrations in active mining ponds. However, the intricate mercury cycle makes it difficult to assess the significance of this finding. While such low-concentration ionic mercury may not be directly toxic, the true danger lies in its conversion to the highly toxic methylmercury (MeHg). The uptake of MeHg by aquatic species is faster than its removal (bioaccumulation), and its concentrations increase with each trophic level (biomagnification) [46,47]. This raises major concerns regarding environmental pollution and human health [8]. As a step towards developing tools for the detection of different chemical species of mercury, we inserted the merB gene into our pUC-Mer-Blue plasmid. Cells transformed with this plasmid produce the organomercurial lyase MerB, which converts organic ethyl- or methylmercury into ionic mercury, thus allowing our original circuit to detect it [14,48,49]. Despite promising initial results, showing the production of blue colour in response to methylmercury standards (Figure S6), further experiments are necessary to develop a reliable methylmercury biosensor.

## 5. Conclusions

In this study, we have illustrated the potential of a simple circuit based on the MerR transcription factor to fulfil practical necessities in low-tech settings. The Mer-RFP fluorescent biosensor allowed detailed dose–response profiling of the circuit and real-time characterization of signal expression and its dependence on growth. The Mer-Blue colorimetric biosensor may provide an economical solution to substantially enhance environmental surveillance in Peru. Despite the ongoing challenge of interference from other pollutants affecting bacterial growth, with additional optimization, our biosensor technology could become an integral component of a comprehensive mercury monitoring strategy.

## Figures and Tables

**Table 1:** Mercury concentrations obtained from the biosensor and ICPMS analysis. The values are the means of three measurements and the corresponding standard deviations are indicated.

			SF-ICP-MS		Mer-RFP		Mer-Blue	
Place	Status	Type of Sample	Std. Dev.	Std. Dev.	[Hg] nM	Std. Dev.	[Hg] nM	Std. Dev.
Laberinto district	Active Pond	Water	0.48	±0.12	<3.2 *		<4.6 *	
		Water–Sediment	2.72	±0.31	<3.2 *		<4.6 *	
	Inactive pond	Water	0.68	±0.18	<3.2 *		<4.6 *	
		Water–Sediment	0.62	±0.04	<3.2 *		<4.6 *	
Isla de los Monos	Active pond	Water	0.60	±0.24	<3.2 *		<4.6 *	
		Water–Sediment	2.37	±0.05	<3.2 *		<4.6 *	
Reaction bucket	Recently mixed	Water	25.13	±0.38	52.3 *	±11.4	<45.9 *	±4.3

* Values are reported as double due to the 2× dilution factor applied to the samples in the culture medium.

## Data Availability

The design files for the custom-built setup used to capture uniform images of culture pellets, including the laser cutting files and the software for color analysis based on Euclidean distance, are available on GitHub: https://github.com/EhbAIGit/PelletCam-OPENBIOLAB-AI (accessed on 20 February 2024).

## Supplementary Materials

The following supporting information can be downloaded at https://www.mdpi.com/article/10.3390/bios14050246/s1: Table S1: Serial dilution of ionic mercury; Table S2: Sequences of the genetic constructs used in this study; Table S3: comparative list of reported WCBs for mercury detection; Figure S1: Testing various bacterial chassis for our biosensor circuit; Figure S2: Bacterial growth analysis of Mer-RFP biosensor; Figure S3: Selectivity of the Mer-Blue WCB; Figure S4: Stability of the Mer-Blue WCB; Figure S5: Exploratory assays of samples from Cajamarca and Iquitos, Peru; Figure S6: Methylmercury assays.

## References

[1] Lamborg C., Bowman K., Hammerschmidt C., Gilmour C., Munson K., Selin N., Tseng C.-M. (2014). Mercury in the Anthropocene Ocean. Oceanog.

[2] Li F., Ma C., Zhang P. (2020). Mercury Deposition, Climate Change and Anthropogenic Activities: A Review. Front. Earth Sci..

[3] Gautam R.K., Sharma S.K., Mahiya S., Chattopadhyaya M.C. (2014). CHAPTER 1. Contamination of Heavy Metals in Aquatic Media: Transport, Toxicity and Technologies for Remediation. Heavy Metals in Water.

[4] Rana M.N., Tangpong J., Rahman M.M. (2018). Toxicodynamics of Lead, Cadmium, Mercury and Arsenic- induced kidney toxicity and treatment strategy: A mini review. Toxicol. Rep..

[5] Yang L., Zhang Y., Wang F., Luo Z., Guo S., Strähle U. (2020). Toxicity of mercury: Molecular evidence. Chemosphere.

[6] Chen C.Y., Driscoll C.T., Eagles-Smith C.A., Eckley C.S., Gay D.A., Hsu-Kim H., Keane S.E., Kirk J.L., Mason R.P., Obrist D. (2018). A Critical Time for Mercury Science to Inform Global Policy. Environ. Sci. Technol..

[7] Gao Y., Shi Z., Long Z., Wu P., Zheng C., Hou X. (2012). Determination and speciation of mercury in environmental and biological samples by analytical atomic spectrometry. Microchem. J..

[8] Wyatt L., Ortiz E.J., Feingold B., Berky A., Diringer S., Morales A.M., Jurado E.R., Hsu-Kim H., Pan W. (2017). Spatial, temporal, and dietary variables associated with elevated mercury exposure in peruvian riverine communities upstream and downstream of artisanal and small-scale gold mining. Int. J. Environ. Res. Public Health.

[9] Lund P.A., Ford S.J., Brown N.L. (1986). Transcriptional regulation of the mercury-resistance genes of transposon Tn501. J. Gen. Microbiol..

[10] Parkhill J., Brown N.L. (1990). Site-specific insertion and deletion mutants in the mer promoter-operator region of Tn501; the nineteen base-pair spacer is essential for normal induction of the promoter by MerR. Nucleic Acids Res..

[11] Wang D., Huang S., Liu P., Liu X., He Y., Chen W., Hu Q., Wei T., Gan J., Ma J. (2016). Structural Analysis of the Hg(II)-Regulatory Protein Tn501 MerR from Pseudomonas aeruginosa. Sci. Rep..

[12] O’Halloran T.V., Frantz B., Shin M.K., Ralston D.M., Wright J.G. (1989). The MerR heavy metal receptor mediates positive activation in a topologically novel transcription complex. Cell.

[13] Brown N.L., Stoyanov J.V., Kidd S.P., Hobman J.L. (2003). The MerR family of transcriptional regulators. FEMS Microbiol. Rev..

[14] Ivask A., Hakkila K., Virta M. (2001). Detection of organomercurials with sensor bacteria. Anal. Chem..

[15] Hansen L.H., Sørensen S.J. (2000). Versatile biosensor vectors for detection and quantification of mercury. FEMS Microbiol. Lett..

[16] Guo M., Du R., Xie Z., He X., Huang K., Luo Y., Xu W. (2019). Using the promoters of MerR family proteins as “rheostats” to engineer whole-cell heavy metal biosensors with adjustable sensitivity. J. Biol. Eng..

[17] Chen S., Chen X., Su H., Guo M., Liu H. (2023). Advances in Synthetic-Biology-Based Whole-Cell Biosensors: Principles, Genetic Modules, and Applications in Food Safety. Int. J. Mol. Sci..

[18] Stocker J., Balluch D., Gsell M., Harms H., Feliciano J., Daunert S., Malik K.A., van der Meer J.R. (2003). Development of a Set of Simple Bacterial Biosensors for Quantitative and Rapid Measurements of Arsenite and Arsenate in Potable Water. Environ. Sci. Technol..

[19] Gui Q., Lawson T., Shan S., Yan L., Liu Y. (2017). The Application of Whole Cell-Based Biosensors for Use in Environmental Analysis and in Medical Diagnostics. Sensors.

[20] Lopreside A., Wan X., Michelini E., Roda A., Wang B. (2019). Comprehensive Profiling of Diverse Genetic Reporters with Application to Whole-Cell and Cell-Free Biosensors. Anal. Chem..

[21] fcoppens Deliberate Release of GMOs’, Belgian Biosafety Server. https://www.biosafety.be/content/deliberate-release-gmos.

[22] Wan X., Volpetti F., Petrova E., French C., Maerkl S.J., Wang B. (2019). Cascaded amplifying circuits enable ultrasensitive cellular sensors for toxic metals. Nat. Chem. Biol..

[23] Scott M., Gunderson C.W., Mateescu E.M., Zhang Z., Hwa T. (2010). Interdependence of cell growth and gene expression: Origins and consequences. Science.

[24] Ahmed F.H., Caputo A.T., French N.G., Peat T.S., Whitfield J., Warden A.C., Newman J., Scott C. (2022). Over the rainbow: Structural characterization of the chromoproteins gfasPurple, amilCP, spisPink and eforRed. Acta Crystallogr. D Struct. Biol..

[25] Campbell R.E., Tour O., Palmer A.E., Steinbach P.A., Baird G.S., Zacharias D.A., Tsien R.Y. (2002). A monomeric red fluorescent protein. Proc. Natl. Acad. Sci. USA.

[26] Liljeruhm J., Funk S.K., Tietscher S., Edlund A.D., Jamal S., Wistrand-Yuen P., Dyrhage K., Gynnå A., Ivermark K., Lövgren J. (2018). Engineering a palette of eukaryotic chromoproteins for bacterial synthetic biology. J. Biol. Eng..

[27] Boyer F., Besson B., Baptist G., Izard J., Pinel C., Ropers D., Geiselmann J., Jong H.D., Cnrs M., Fourier U.J. (2010). WellReader: A MATLAB program for the analysis of fluorescence and luminescence reporter gene data. Bioinformatics.

[28] Rogers J.K., Guzman C.D., Taylor N.D., Raman S., Anderson K., Church G.M. (2015). Synthetic biosensors for precise gene control and real-time monitoring of metabolites. Nucleic Acids Res..

[29] Du R., Guo M., He X., Huang K., Luo Y., Xu W. (2019). Feedback regulation mode of gene circuits directly affects the detection range and sensitivity of lead and mercury microbial biosensors. Anal. Chim. Acta.

[30] Prabowo B.A., Cabral P.D., Freitas P., Fernandes E. (2021). The Challenges of Developing Biosensors for Clinical Assessment: A Review. Chemosensors.

[31] Wang D., Zheng Y., Fan X., Xu L., Pang T., Liu T., Liang L., Huang S., Xiao Q., Al W.E.T. (2020). Visual detection of Hg2+ by manipulation of pyocyanin biosynthesis through the Hg2+ dependent transcriptional activator MerR in microbial cells. J. Biosci. Bioeng..

[32] Brophy J.A.N., Voigt C.A. (2014). Principles of genetic circuit design. Nat. Methods.

[33] Food and Agriculture Organization of the United Nations (FAO) and World Health Organization (WHO) (2019). General Standard for Contaminants and Toxins in Food and FeedCodex Alimentariusen.pdf. International Food Standards. https://www.fao.org/fao-who-codexalimentarius/sh-proxy/en/?lnk=1&url=https%253A%252F%252Fworkspace.fao.org%252Fsites%252Fcodex%252FStandards%252FCXS%2B193-1995%252FCXS_193e.pdf.

[34] Wang D., Zheng Y., Xu L., Fan X., Wei N., Jin N., Huang S., Xiao Q., Wu Z. (2019). Engineered cells for selective detection and remediation of Hg2+ based on transcription factor MerR regulated cell surface displayed systems. Biochem. Eng. J..

[35] Guo Y., Hui C.y., Liu L., Chen M.P., Huang H.Y. (2021). Development of a bioavailable Hg(II) sensing system based on MerR-regulated visual pigment biosynthesis. Sci. Rep..

[36] Cai S., Shen Y., Zou Y., Sun P., Wei W., Zhao J., Zhang C. (2018). Engineering highly sensitive whole-cell mercury biosensors based on positive feedback loops from quorum-sensing systems. Analyst.

[37] Din G., Hasan F., Conway M., Denney B., Ripp S., Shah A.A. (2019). Engineering a bioluminescent bioreporter from an environmentally sourced mercury-resistant Enterobacter cloacae strain for the detection of bioavailable mercury. J. Appl. Microbiol..

[38] Guo M., Wang J., Du R., Liu Y., Chi J., He X., Huang K., Luo Y., Xu W. (2020). A test strip platform based on a whole-cell microbial biosensor for simultaneous on-site detection of total inorganic mercury pollutants in cosmetics without the need for predigestion. Biosens. Bioelectron..

[39] Mahbub K.R., Krishnan K., Naidu R., Megharaj M. (2017). Development of a whole cell biosensor for the detection of inorganic mercury. Environ. Technol. Innov..

[40] Priyadarshi H., Alam A., Gireesh-Babu P., Das R., Kishore P., Kumar S., Chaudhari A. (2012). A GFP-based bacterial biosensor with chromosomally integrated sensing cassette for quantitative detection of Hg(II) in environment. J. Environ. Sci. China.

[41] Wei H., Cheng H., Ting M., Wen-Hui Z., Xian-Gui L. (2010). A chromosomally based luminescent bioassay for mercury detection in red soil of China. Appl. Microbiol. Biotechnol..

[42] Zhang N.-X., Guo Y., Li H., Yang X.-Q., Gao C.-X., Hui C.-Y. (2021). Versatile artificial mer operons in Escherichia coli towards whole cell biosensing and adsorption of mercury. PLoS ONE.

[43] Lee K.H., Kim D.M. (2019). In vitro use of cellular synthetic machinery for biosensing applications. Front. Pharmacol..

[44] Noireaux V., Liu A.P. (2020). The New Age of Cell-Free Biology. Annu. Rev. Biomed. Eng..

[45] Guzman-Chavez F., Arce A., Adhikari A., Vadhin S., Pedroza-Garcia J.A., Gandini C., Ajioka J.W., Molloy J., Sanchez-Nieto S., Varner J.D. (2022). Constructing Cell-Free Expression Systems for Low-Cost Access. ACS Synth. Biol..

[46] Alexandre J.P., Tamar B. (2013). Cracking the Mercury Methylation Code. Science.

[47] Diringer S.E., Feingold B.J., Ortiz E.J., Gallis J.A., Araújo-Flores J.M., Berky A., Pan W.K.Y., Hsu-Kim H. (2015). River transport of mercury from artisanal and small-scale gold mining and risks for dietary mercury exposure in Madre de Dios, Peru. Environ. Sci. Process. Impacts.

[48] Griffin H.G., Foster T.J., Silver S., Misra T.K. (1987). Cloning and DNA sequence of the mercuric- and organomercurial-resistance determinants of plasmid pDU1358. Proc. Natl. Acad. Sci. USA.

[49] Nucifora G., Chu L., Silver S., Misra T.K. (1989). Mercury operon regulation by the merR gene of the organomercurial resistance system of plasmid pDU1358. J. Bacteriol..

