# Supplementary material for: Highly Sensitive Whole-Cell Mercury Biosensors for Environmental Monitoring
Source: Biosensors (Basel). 2024 May 13;14(5):246. doi: 10.3390/bios14050246 (PMC11117708; doi:10.3390/bios14050246)
Supplement: Supplementary file 1 [file biosensors-14-00246-s001.zip › biosensors-2955775-supplementary.pdf]

SUPPORTING INFORMATION

Highly Sensitive Whole-Cell Mercury Biosensors for Environmental Monitoring

Tube numbering	1	2	3	4	5	6	7	8
Intended $HgBr_2$ concentration	$2\ mM$	$1\ mM$	$500\ \mu M$	$250\ \mu M$	$125\ \mu M$	$50\ \mu M$	$25\ \mu M$	$10\ \mu M$
Volume from preceding tube	-	$500\ \mu l$	$500\ \mu l$	$500\ \mu l$	$500\ \mu l$	$400\ \mu l$	$500\ \mu l$	$400\ \mu l$
ddH_2O	-	$500\ \mu l$	$500\ \mu l$	$500\ \mu l$	$500\ \mu l$	$600\ \mu l$	$500\ \mu l$	$600\ \mu l$
Total volume	$1000\ \mu l$	$1000\ \mu l$	$1000\ \mu l$	$1000\ \mu l$	$1000\ \mu l$	$1000\ \mu l$	$1000\ \mu l$	$1000\ \mu l$

Tube numbering	9	10	11	12	13	14	15
Intended $HgBr_2$ concentration	$5\ \mu M$	$2\ \mu M$	$1\ \mu M$	$400\ nM$	$200\ nM$	$80\ nM$	$40\ nM$
Volume from preceding tube	$500\ \mu l$	$400\ \mu l$	$500\ \mu l$	$400\ \mu l$	$500\ \mu l$	$400\ \mu l$	$500\ \mu l$
ddH_2O	$500\ \mu l$	$600\ \mu l$	$500\ \mu l$	$600\ \mu l$	$500\ \mu l$	$600\ \mu l$	$500\ \mu l$
Total volume	$1000\ \mu l$	$1000\ \mu l$	$1000\ \mu l$	$1000\ \mu l$	$1000\ \mu l$	$1000\ \mu l$	$1000\ \mu l$

Table S1. Serial dilution of ionic mercury. A stock of 2 mM $HgBr_2$ was prepared by diluting solid powder in ddH_2O. Serial dilutions were prepared prior to each assay and stored in a refrigerated environment (4°C). When applied to microcultures, 5 µL of $HgBr_2$ solutions were diluted 1/40 in a total volume of 200 µL of WCB. When applied to minicultures, 10 µL of $HgBr_2$ solutions were diluted 1/1000 in a total volume of 10 mL of WCB.

**Name**	DNA sequence (5´-3)
**Lambda TR2 terminator**	(PfoI)gtcacacgactaggacttcagatgggagtgagctgataccgcccatggtcgccgcagccgaacgagccgagcgcaga<br>**GTTAGTTTTTCATGACTTCCCTCTCCCCCAAATAAAAAGGCCTGCGATTACCAGCAGGCCTGTT**<br>**ATTAGCTCAGTAATGTAGATGGTCAT**tggtgacgtggcccactggc (XhoI)
**merR gene**	(XhoI)tggcatcgtgcgtcacag**CTACGGCATAGCTGATCCCGCCAGGCTAGCCCCTCCTTGAAGCGAC**<br>**GCGATCAATGGACACGAAACATTACCGCGACGTGCATGACAAGCACATACCAACTCGCTCAAG**<br>**ACAGCCTCCATACGGGCCAGATCGGCCATTTTTTCGCGCACGTCTTTCAATTTATGCTCAGCTAA**<br>**AGAGCTAGCCTCTTCGCAATGCGTGCCGTCTTCTAAGCGCAGAAGTTCCGCAATCTCGTCAAGC**<br>**GAGAAACCAAGGCGCTGCGCCGACTTGACAAAACGGACGCGAGTAACGTCAGCTTCGCCATA**<br>**ACGACGGATGGAACCGTAAGGCTTATCGGGCTCAAGCAGAAGTCCTTTACGCTGATAGAAACG**<br>**GATTGTCTCAACATTTACGCCCGCCGCTTTAGCGAATACGCCAATCGTCAAATTCTCCAAATTAT**<br>**TTTCCAT**ctagatttctcctctttactctagtatg(SacI)
**429 promoter**	**(**SacI**)**Tgtga**TTACCAACAACATACGAGCCGGAAGCATAAAGTGTAATAAACA**ggattacggattcact<br>ggccgtactagtcgttttacaacgtcgtgactccgaaaaccctggcgttacccaacttaatcgccttgcagcacatccccctttcg<br>gctggcgtaatagcgacagaggcccgcaccgcgcccttcgcaacagttggcagcctgacatggcgaatggacgctttgcctgg<br>tttccggcaccagaagcggtgccggaactggcagagtgcatcttccgccgatacttgtcgtcgtcctcactcaaactggcagatg<br>cacggttacgatgcgcccatctacaccaacgtaacctatcccattacggtcaatccgccgtttgttccgcagcagaatcacgacg<br>ggttgttactcgctcacatttaatgttgatgaaagaggctggctaggaaggccagacgcgaattatttttgatggcgtatggaat<br>tacgttatcgactgtcacgcaatgcttctgcgtcaggcagccatcggaagctgtggtatggctgtcgcagtcgtaaatcagtctg<br>cata**(**BamHI**)**<br><br>**Parts of 429 promoter**<br>**-35 site: tgtaat**<br>**-10 site: aacata**
**PTn501-amilCP**	(BamHI)attcgtgtcgctcaaggcga**ATCGCTTGACTCCGTACATGAGTACGGAAGTAAGGTTACGCTA**<br>**TCCAATTTCAATTCGAAAGGACAAGCATATGAGCGTGATTGCAAAGCAGATGACCTATAAAGT**<br>**TTATATGAGCGGCACCGTGAACGGCCATTATTTTGAAGTTGAAGGTGATGGTAAAGGCAAACC**<br>**GTATGAAGGTGAACAGACCGTTAAACTGACCGTTACCAAAGGTGGTCCGCTGCCGTTTGCATG**<br>**GGATATTCTGAGTCCGCAGTGTCAGTATGGTAGCATTCCGTTTACCAAATATCCGGAAGATATC**<br>**CCGGATTATGTGAAACAGAGCTTTCCGGAAGGTTATACCTGGGAACGTATTATGAATTTTGAA**<br>**GATGGTGCCGTTTGTACCGTTAGCAATGATAGCAGCATTCAGGGTAATTGCTTTATCTACCACG**<br>**TGAAATTTAGCGGTCTGAATTTTCCGCCTAATGGTCCGGTTATGCAGAAAAAAACCCAAGGTTG**<br>**GGAACCGAATACCGAACGTCTGTTTGCACGTGATGGTATGCTGCTGGGTAATAACTTTATGGC**<br>**ACTGAAACTGGAAGGTGGTGGTCATTATCTGTGTGAGTTCAAAACCACCTACAAAGCCAAAAA**<br>**ACCGGTTAAAATGCCTGGCTATCATTATGTGGATCGTAAACTGGATGTGACCAACCACAATAA**<br>**AGATTACACCAGCGTTGAACAGTGCGAAATTAGCATTGCACGTAAACCGGTTGTTGCCTAA**taat<br>act(NotI)<br><br>**Parts of PTn501 promoter**<br>**operator mer site:** cgcttgactccgtacatgagtacggaagtaa<br>**-35 site:** ttgact<br>**-10 site:** taaggt

**BBa_B0014 terminator**	**(NotI)**agag**TCACACTGGCTCACCTTCGGGTGGGCCTTTCTGCGTTTATATACTAGAGAGAGAAT**<br>**ATAAAAAGCCAGATTATTAATCCGGCTTTTTATTATTT**ccggtcagtgagcgagggtaccgaagcgcaaga<br>gcccctctggagctgatcttgtgtgtag**(PscI)**
**pUC57 vector**	(PscI)gagcaaaaggccagcaaaaggccaggaaccgtaaaaaggccgcgttgctggcgtttttccataggctccgcccccctg<br>acgagcatcacaaaaatcgacgctcaagtcagaggtggcgaaacccgacaggactataaagataccaggcgtttccccctgg<br>aagctccctcgtgcgctctcctgttccgaccctgccgcttaccggatacctgtccgcctttctcccttcgggaagcgtggcgctttc<br>tcatagctcacgctgtaggtatctcagttcggtgtaggtcgttcgctccaagctgggctgtgtgcacgaaccccccgttcagcccg<br>accgctgcgccttatccggtaactatcgtcttgagtccaacccggtaagacacgacttatcgccactggcagcagccactggta<br>acaggattagcagagcgaggtatgtaggcggtgctacagagttcttgaagtggtggcctaactacggctacactagaagaaca<br>gtatttggtatctgcgctctgctgaagccagttaccttcggaaaaagagttggtagctcttgatccggcaaacaaaccaccgctg<br>gtagcggtggtttttttgtttgcaagcagcagattacgcgcagaaaaaaaggatctcaagaagatcctttgatcttttctacggg<br>gtctgacgctcagtggaacgaaaactcacgttaagggattttggtcatgagattatcaaaaaggatcttcacctagatccttttta<br>aattaaaaatgaagttttaaatcaatctaaagtatatatgagtaaacttggtctgacagttaccaatgcttaatcagtgaggcac<br>ctatctcagcgatctgtctatttcgttcatccatagttgcctgactccccgtcgtgtagataactacgatacgggagggcttaccat<br>ctggccccagtgctgcaatgataccgcgagacccacgctcaccggctccagatttatcagcaataaaccagccagccggaagg<br>gccgagcgcagaagtggtcctgcaactttatccgcctccatccagtctattaattgttgccgggaagctagagtaagtagttcgc<br>cagttaatagtttgcgcaacgttgttgccattgctacaggcatcgtggtgtcacgctcgtcgtttggtatggcttcattcagctccg<br>gttcccaacgatcaaggcgagttacatgatcccccatgttgtgcaaaaaagcggttagctccttcggtcctccgatcgttgtcag<br>aagtaagttggccgcagtgttatcactcatggttatggcagcactgcataattctcttactgtcatgccatccgtaagatgcttttc<br>tgtgactggtgagtactcaaccaagtcattctgagaatagtgtatgcggcgaccgagttgctcttgcccggcgtcaatacgggat<br>aataccgcgccacatagcagaactttaaaagtgctcatcattggaaaacgttcttcggggcgaaaactctcaaggatcttaccg<br>ctgttgagatccagttcgatgtaacccactcgtgcacccaactgatcttcagcatcttttactttcaccagcgtttctgggtgagca<br>aaaacaggaaggcaaaatgccgcaaaaaagggaataagggcgacacggaaatgttgaatactcatactcttcctttttcaata<br>ttattgaagcatttatcagggttattgtctcatgagcggatacatatttgaatgtatttagaaaaataaacaaataggggttccgc<br>gcacatttccccgaaaagtgccacctgacgtctaagaaaccattattatcatgacattaacctataaaaataggcgtatcacga<br>ggccctttcgtctcgcgcgtttcggtgatgacggtgaaaacctctgacacatgcagc(PfoI)
**Gene block rfp gene**	catcgcacctacatctgtattaacgaagcggtgtgggcgcat**ATGGCAAGCAGCGAAGATGTGATCAAAGAAT**<br>**TTATGCGTTTCAAGGTGCGTATGGAAGGTAGCGTTAATGGTCATGAATTTGAAATTGAAGGTG**<br>**AAGGCGAAGGTCGTCCGTATGAAGGCACCCAGACCGCAAAACTGAAAGTTACCAAAGGTGGT**<br>**CCGCTGCCGTTTGCATGGGATATTCTGAGTCCGCAGTTTCAGTATGGTAGCAAAGCATACGTTA**<br>**AACATCCGGCAGATATCCCGGATTATCTGAAACTGAGCTTTCCGGAAGGTTTTAAATGGGAAC**<br>**GTGTGATGAATTTTGAAGATGGTGGTGTTGTTACCGTTACACAGGATAGCAGCCTGCAGGATG**<br>**GTGAATTTATCTATAAAGTTAAACTGCGTGGCACGAATTTTCCGAGTGATGGTCCGGTTATGCA**<br>**GAAAAAAACaATGGGTTGGGAAGCAAGCACCGAACGTATGTATCCGGAAGATGGCGCACTGA**<br>**AAGGTGAAATCAAAATGCGTCTGAAGCTGAAAGATGGCGGTCATTATGATGCAGAAGTTAAA**<br>**ACCACCTACATGGCCAAAAAACCGGTTCAGCTGCCTGGTGCATATAAAACCGATATTAAACTG**<br>**GATATCACCAGCCACAACGAGGATTATACCATTGTTGAACAGTATGAACGTGCAGAAGGTCGC**<br>**CATAGTACCGGTGCATAA**taagcggccgcgggtcgttagatagccgttatgtcat

Table S2. Sequences of the genetic constructs used in this study. The DNA sequence in capital letters corresponds to the circuit part indicated in the table. The RBS_{merR} sequence (Bba_K1758342) is underlined in blue. The amilCP and rfp genes are noted in blue and red text, respectively, and were flanked by NdeI (underlined in dark green) and NotI (underlined in purple).

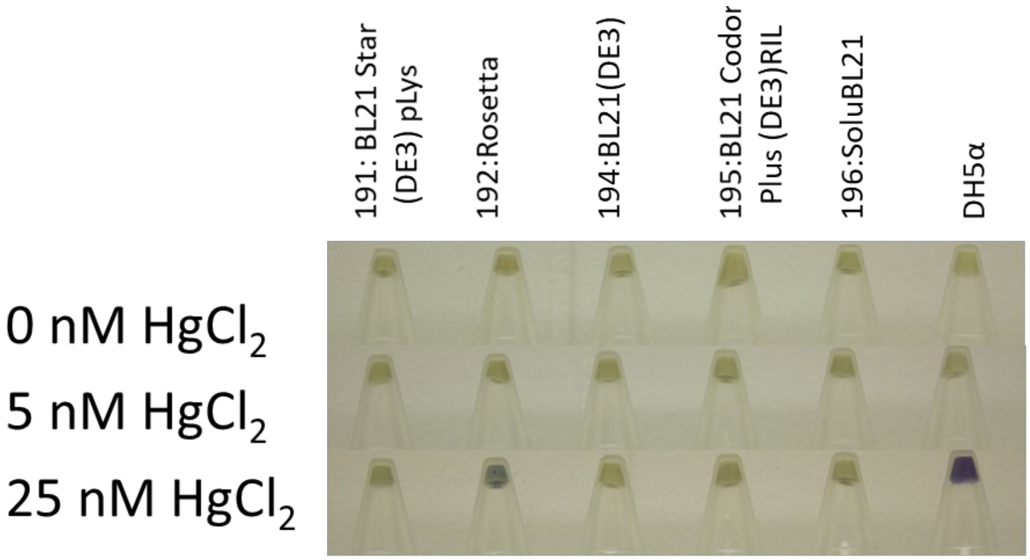

Figure S1: Testing various bacterial chassis for our biosensor circuit. Minicultures were prepared and pelleted as described in the Materials and Methods section. Under similar conditions, cultures of DH5α cells show higher, more conspicuous signal.

A)

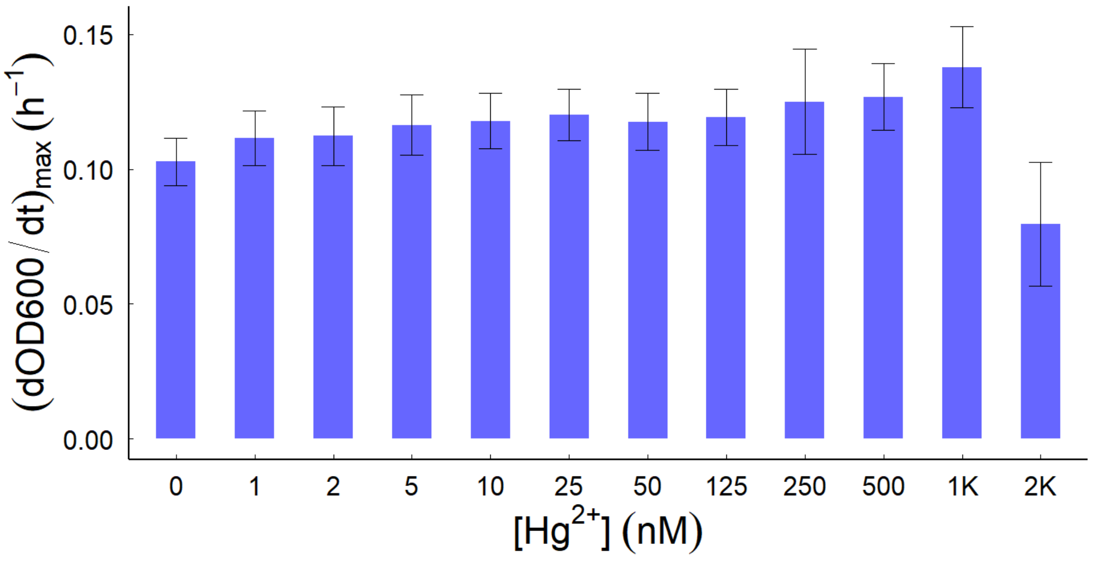

B)

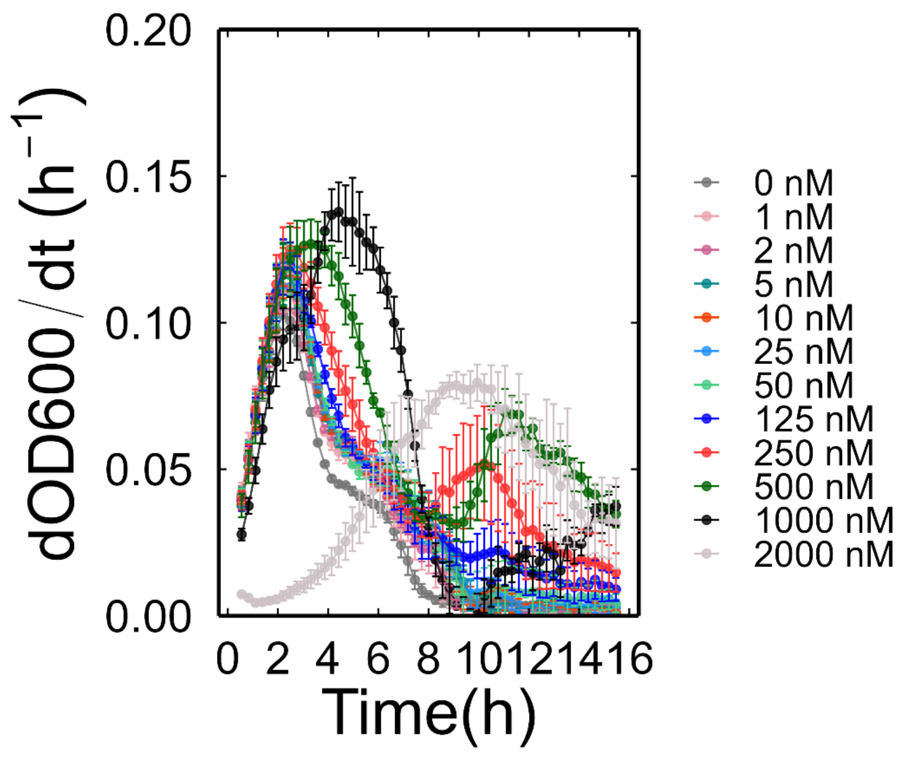

Figure S2. Bacterial growth analysis of Mer-RFP biosensor. (A) Maximum growth rate of the biosensor in response to various concentrations of $HgBr_2$. Kruskal-Wallis One-Way ANOVA revealed a significantly reduced growth rate for the culture exposed to 2,000 nM Hg^{2+} compared to other conditions ($p < 0.001$). (B) Continuous calculation of growth rates for the Mer-RFP biosensor. Peaks were identified and plotted in the previous graph within the 2-5 hour interval, except for the highest concentration, which was observed at 10 hours. Error bars represent the standard error from three independent replicates.

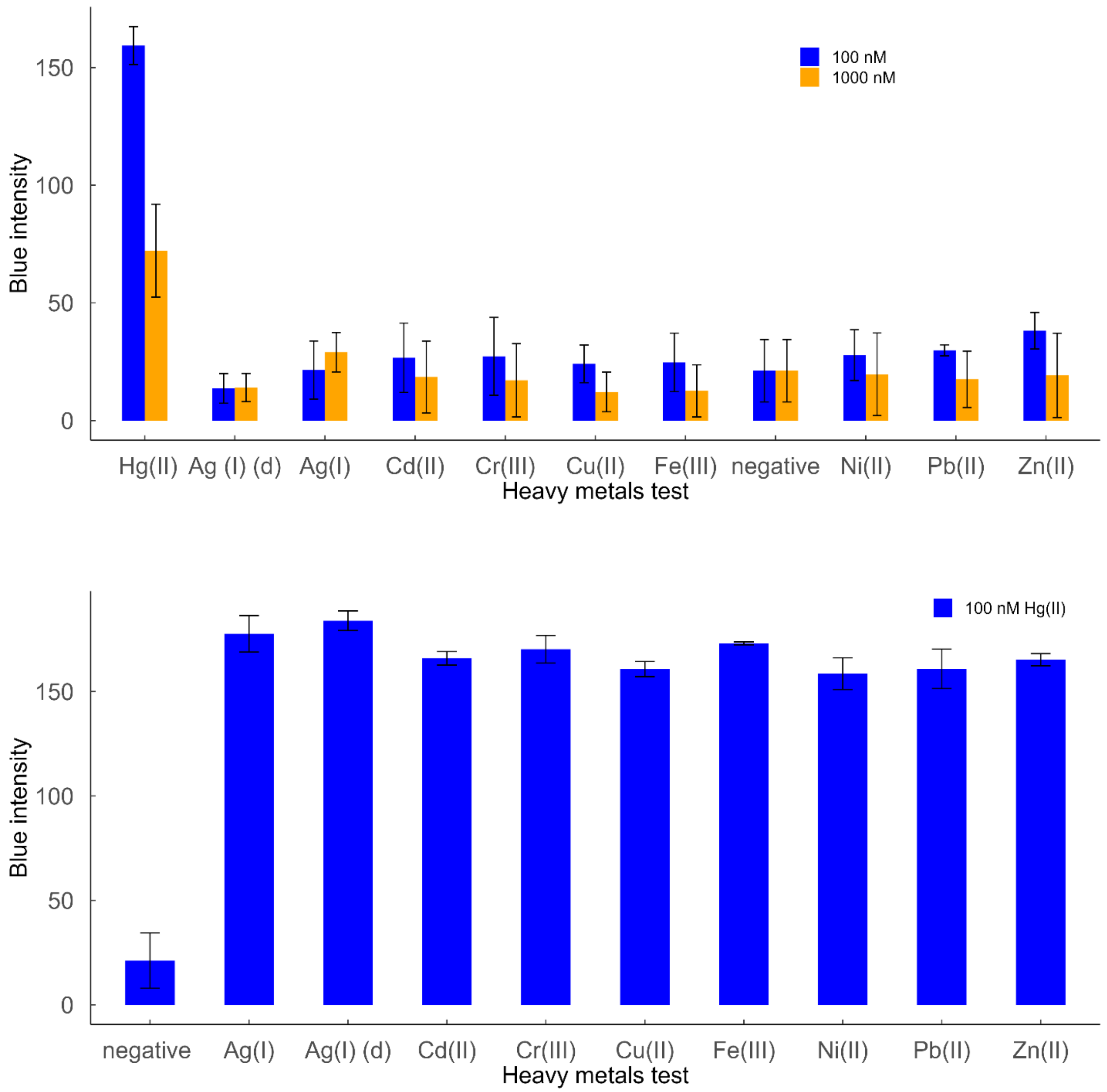

Figure S3. Selectivity of the Mer-Blue biosensor. Top panel, response in the presence of 100 nM of various metal ions. Bottom panel, response to 100 nM Hg^{2+} in the presence of additional 100 nM of various metal ions.

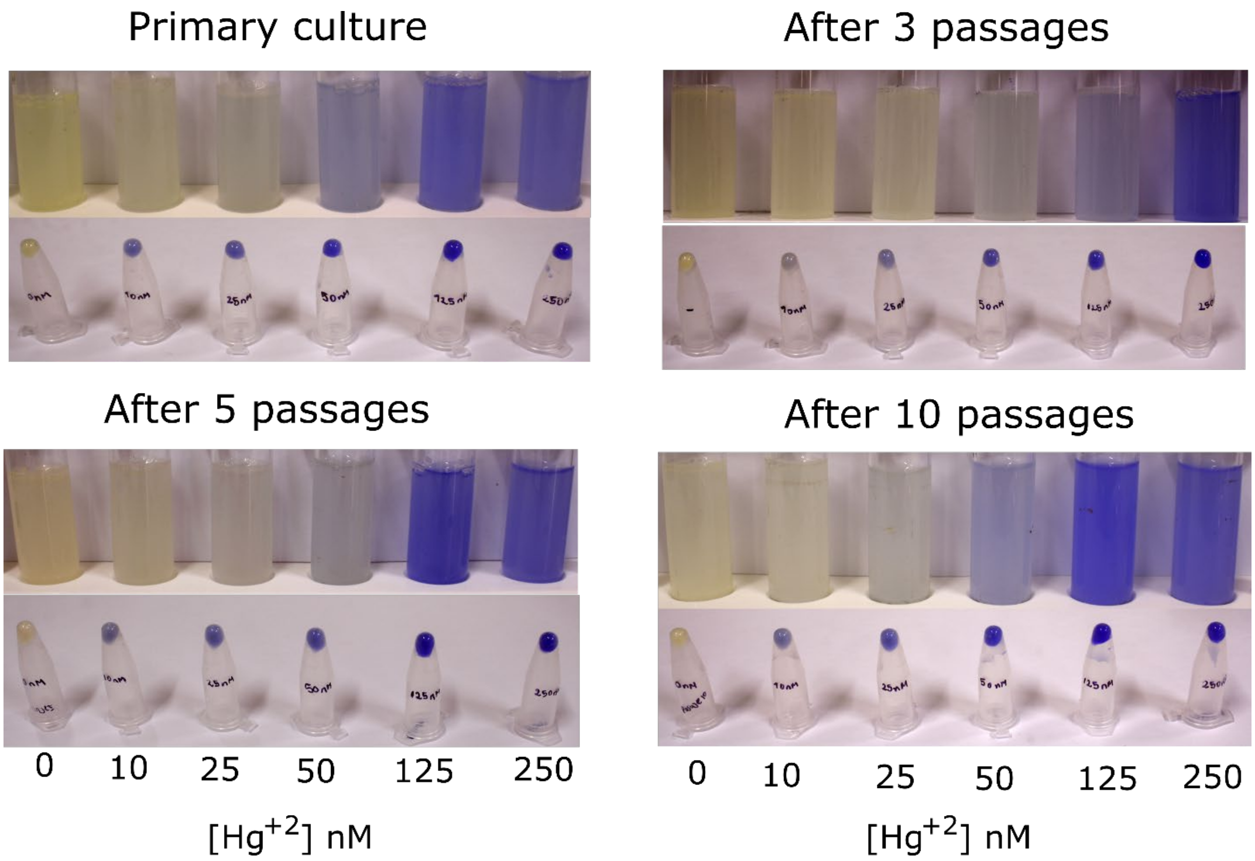

Figure S4. Stability of the Mer-Blue Biosensor. Primary culture: Freshly transformed DH5α cells were cultured from a single colony on solid agar to an overnight liquid culture, which was then utilized to initiate the biosensor test, as outlined in the Materials and Methods section. After N passages: An overnight culture was diluted 1/20 in fresh medium containing ampicillin (AMP) and incubated for 24 hours; this process was repeated N times. Subsequently, the biosensor test was performed as described previously.

Samples from Cajamarca

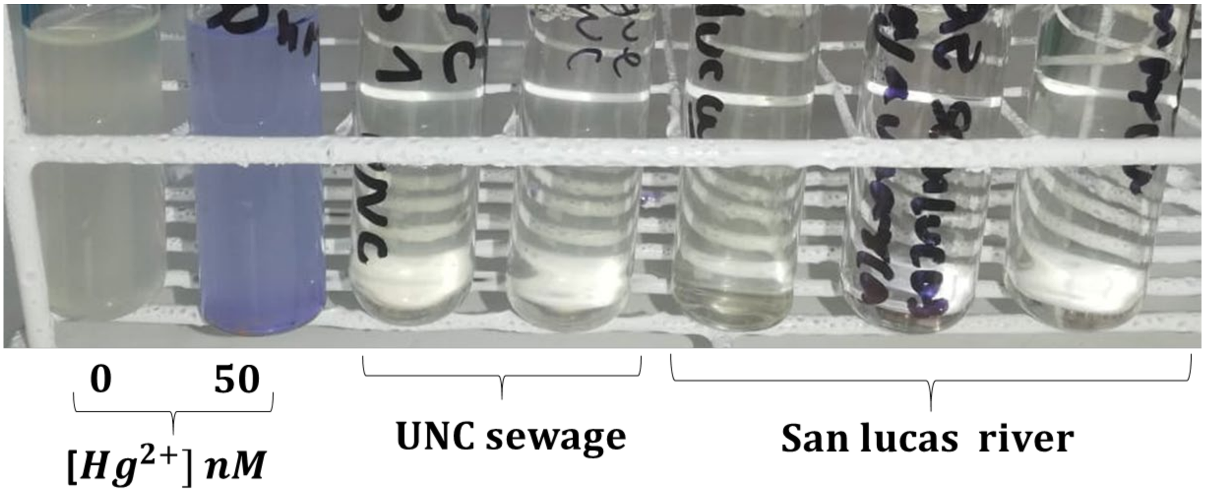

Samples from Iquitos

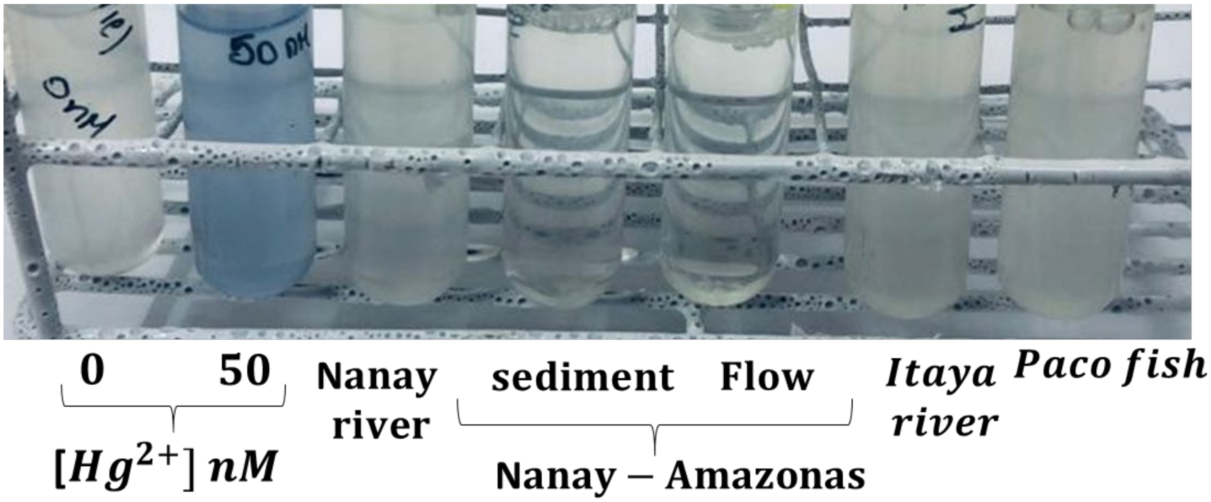

Figure S5. Exploratory Assays of Samples from Cajamarca and Iquitos, Peru. Controls of 0 and 50 nM $HgBr_2$ were included in each test, depicted on the left side of the image. Cajamarca samples were collected from wastewater from the National University of Cajamarca (UNC) and from the San Lucas River. Iquitos samples were collected from the Nanay and Itaya rivers, as well as from the confluence of the Nanay and Amazon rivers (Nanay-Amazonas). All liquid samples were passed through a 0.22 µm syringe filter for sterilization. A small solid sample was taken from fish from a local market (Paco fish), minced with water, and filtered through a 0.22 µm syringe filter. The samples were analysed according to the protocol outlined in the Materials and Methods section. Note: The pH levels of samples from rivers ranged between 6 and 7.

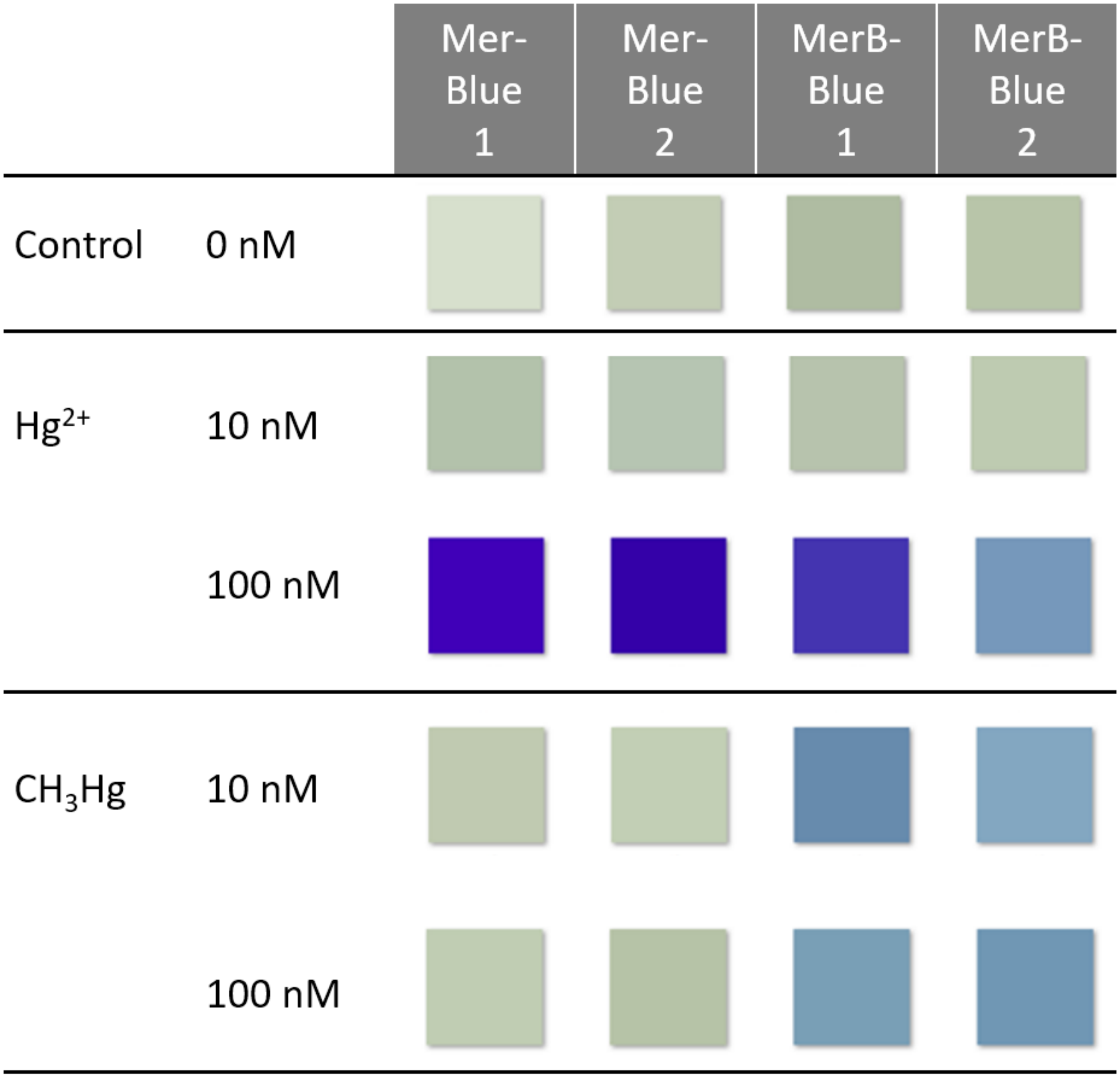

Figure S6. Methylmercury assays. DH5α cells were transformed with pUC-Mer-Blue (Mer-Blue) or with a modified version of this plasmid with the additional coding for the constitutive expression of organomercurial lyase (MerB-Blue). Two single colonies of each biosensor were picked and grown to seed minicultures with the indicated concentrations of Hg^{2+} or methylmercury, following the protocol outlined in the Materials and Methods section. The panels display the blue intensity of 9 pixels located in the centre of the pellets collected from each miniculture.

No	$Host\ cells$	$Biosensing\ modules$	$linear\ range\ (nM)$	$LOD\ (nM)$	$Specifcity$	Ref
1	*E. coli* DH5α	zntR-Pznt-egfp-hj1	2,500-7,500	1000	Hg^{2+}, Cd^{2+}	(1)
2	*E. coli* JM109	chromosomally based merRPmer-gfp	100-1,700	-	Hg^{2+}	(2)
3	*E. coli* TOP10	merR-Pmer-mcherry	6,250-200,000	-	Hg^{2+}	(3)
4	*E. coli* DH5α	merR-Pmer-rfp	50–10,000		Hg^{2+}	(4)
5	*E. coli* DH5α	mer-rfp quorum-sensing system	10–250	10	Hg^{2+}	(5)
6	*Sphingobium* SA2	chromosomally based partial merA-gfp	20–40	-	Hg^{2+}	(6)
7	*Pseudomonas putida*	chromosomally based merRPmer-egfp	200–1400	-	Hg^{2+}	(7)
8	*Enterobacter cloacae*	merR-Pmer-lux	2–7980	1	Hg^{2+}	(8)
9	*Pseudomonas aeruginosa* PAO1	merR-Pmer-phzM-Pmer-phzS-pAK1900	25-1,000	10	Hg^{2+}	(9)
10	*E. coli* DH5α	merR mer-ompA-mcherry-pSB1A2	0.1-100	0.1	Hg^{2+}	(10)
11	*E. coli* DH5α	merR558	1-100	1	Hg^{2+}, Cd^{2+}	(11)
12	*E. coli* TOP10	exponential phase	780–12,500	390	Hg^{2+}	(12)
		merR-Pmer-vioABCDE	-	6	Hg^{2+}	
13	*E. coli* TOP10	J109-$P_{merT} - gfp$	-	1.1	Hg^{2+}	(13)
		3-layer (RS-RinA-E11)(4A3)+ $P_{e11} - gfp(1K3)$	-	0.037	Hg^{2+}	
14	*E. coli* DH5α	Mer-Blue	2.3-125	2.3	Hg^{2+}	This study
		Mer-RFP	1.6-1,000	1.6	Hg^{2+}	

Table S3. Reported whole-cell biosensors for ionic mercury detection.

References

1. Kim H, Lee W, Yoon Y. Heavy metal(loid) biosensor based on split-enhanced green fluorescent protein: development and characterization. Appl Microbiol Biotechnol. 2019;103(15):6345–52.

2. Priyadarshi H, Alam A, Gireesh-Babu P, Das R, Kishore P, Kumar S, et al. A GFP-based bacterial biosensor with chromosomally integrated sensing cassette for quantitative detection of Hg(II) in environment. J Environ Sci [Internet]. 2012;24(5):963–8. Available from: http://dx.doi.org/10.1016/S1001-0742(11)60820-6

3. Zhang NX, Guo Y, Li H, Yang XQ, Gao CX, Hui CY. Versatile artificial mer operons in Escherichia coli towards whole cell biosensing and adsorption of mercury. PLoS One [Internet]. 2021;16(5 May):1–14. Available from: http://dx.doi.org/10.1371/journal.pone.0252190

4. Guo M, Wang J, Du R, Liu Y, Chi J, He X, et al. A test strip platform based on a whole-cell microbial biosensor for simultaneous on-site detection of total inorganic mercury pollutants in cosmetics without the need for predigestion. Biosens Bioelectron [Internet]. 2020;150(November):111899. Available from: https://doi.org/10.1016/j.bios.2019.111899

5. Paquin F, Rivnay J, Salleo A, Stingelin N, Silva C. Engineered Highly Sensitive Whole-Cell Mercury Biosensors Based on Positive Feedback Loop from Quorum-Sensing System. R Soc Chem [Internet]. 2017;3. Available from: http://xlink.rsc.org/?DOI=C5TC02043C

6. Mahbub KR, Krishnan K, Naidu R, Megharaj M. Development of a whole cell biosensor for the detection of inorganic mercury. Environ Technol Innov [Internet]. 2017;8:64–70. Available from: http://dx.doi.org/10.1016/j.eti.2017.04.003

7. Xian-Gui L, Wei H, Cheng H, Ting M, Wen-Hui Z. A chromosomally based luminescent bioassay for mercury detection in red soil of China. Appl Microbiol Biotechnol. 2010;87(3):981–9.

8. Din G, Hasan F, Conway M, Denney B, Ripp S, Shah AA. Engineering a bioluminescent bioreporter from an environmentally sourced mercury-resistant Enterobacter cloacae strain for the detection of bioavailable mercury. J Appl Microbiol. 2019;127(4):1125–34.

9. Wang D, Zheng Y, Fan X, Xu L, Pang T, Liu T, et al. Visual detection of Hg2+ by manipulation of pyocyanin biosynthesis through the Hg2+ dependent transcriptional activator MerR in microbial cells. J Biosci Bioeng. 2019;

10. Wang D, Zheng Y, Xu L, Fan X, Wei N, Jin N, et al. Engineered cells for selective detection and remediation of Hg2+ based on transcription factor MerR regulated cell surface displayed systems. Biochem Eng J [Internet]. 2019;150(April):107289. Available from: https://doi.org/10.1016/j.bej.2019.107289

11. Du R, Guo M, He X, Huang K, Luo Y, Xu W. Feedback regulation mode of gene circuits directly affects the detection range and sensitivity of lead and mercury microbial biosensors. Anal Chim Acta [Internet]. 2019;1084(17):85–92. Available from: https://doi.org/10.1016/j.aca.2019.08.006

12. Guo Y, Hui C ye, Liu L, Chen M peng, Huang H ying. Development of a bioavailable Hg(II) sensing system based on MerR-regulated visual pigment biosynthesis. Sci Rep [Internet]. 2021;11(1):1–14. Available from: https://doi.org/10.1038/s41598-021-92878-6

13. Wan X, Volpetti F, Petrova E, French C, Maerkl SJ, Wang B. Cascaded amplifying circuits enable ultrasensitive cellular sensors for toxic metals. Nat Chem Biol [Internet]. 2019;15(5):540–8. Available from: http://dx.doi.org/10.1038/s41589-019-0244-3
